# Health Implications of High Dietary Omega-6 Polyunsaturated Fatty Acids

**DOI:** 10.1155/2012/539426

**Published:** 2012-04-05

**Authors:** E. Patterson, R. Wall, G. F. Fitzgerald, R. P. Ross, C. Stanton

**Affiliations:** ^1^Alimentary Pharmabiotic Centre, Biosciences Institute, County Cork, Ireland; ^2^Teagasc Food Research Centre, Biosciences Department, Moorepark, Fermoy, County Cork, Ireland; ^3^Department of Microbiology, University College Cork, County Cork, Ireland

## Abstract

Omega-6 (n-6) polyunsaturated fatty acids (PUFA) (e.g., arachidonic acid (AA)) and omega-3 (n-3) PUFA (e.g., eicosapentaenoic acid (EPA)) are precursors to potent lipid mediator signalling molecules, termed “eicosanoids,” which have important roles in the regulation of inflammation. In general, eicosanoids derived from n-6 PUFA are proinflammatory while eicosanoids derived from n-3 PUFA are anti-inflammatory. Dietary changes over the past few decades in the intake of n-6 and n-3 PUFA show striking increases in the (n-6) to (n-3) ratio (~15 : 1), which are associated with greater metabolism of the n-6 PUFA compared with n-3 PUFA. Coinciding with this increase in the ratio of (n-6) : (n-3) PUFA are increases in chronic inflammatory diseases such as nonalcoholic fatty liver disease (NAFLD), cardiovascular disease, obesity, inflammatory bowel disease (IBD), rheumatoid arthritis, and Alzheimer's disease (AD). By increasing the ratio of (n-3) : (n-6) PUFA in the Western diet, reductions may be achieved in the incidence of these chronic inflammatory diseases.

## 1. Introduction

Fatty acids are hydrocarbon chains with a carboxyl group at one end and a methyl group at the other. The biological reactivity of fatty acids is defined by the length of the carbon chain and by both the number and position of any double bonds present. While saturated fatty acids do not contain double bonds within the acyl chain, unsaturated fatty acids contain at least one double bond. When two or more double bonds are present, unsaturated fatty acids are referred to as PUFA [1]. There are two families of PUFA, and they are classified as omega-3 (n-3) and omega-6 (n-6) based on the location of the last double bond relative to the terminal methyl end of the molecule [2]. The human body can produce all but two of the fatty acids it requires. Linoleic acid (LA, C18:2n-6) (precursor to the n-6 series of fatty acids) and *α*-linolenic acid (ALA, C18:3n-3) (precursor to the n-3 series of fatty acids) are the simplest members of each family of PUFA and are termed essential fatty acids as the body cannot synthesise these. PUFA regulate a wide variety of biological functions, depending on the location of the last double bond, which range from blood pressure and blood clotting to the correct development and functioning of the brain and nervous system [2]. In addition, lipid mediators generated from long-chain (LC-) PUFA (arachidonic acid (AA) in the n-6 series and eicosapentaenoic acid (EPA) and docosahexaenoic acid (DHA) in the n-3 series) have important roles in immune regulation and inflammation [3]. The main dietary sources of LA include plant oils such as sunflower, safflower, and corn oils (Table 1), but they are also present in cereals, animal fat, and wholegrain bread. Rich dietary sources of ALA include green leafy vegetables, flaxseed, and rapeseed oils [2] (Table 1).

Over the last few decades, extreme qualitative nutritional changes have taken place with increased levels of fatty acid consumption [4]. Today, industrialised societies are characterised by an increase in saturated fat, omega 6 PUFA, and trans fatty acid intake, as well as an overall decrease in omega-3 PUFA intake [5]. Fatty acids now represent 28–42% of total energy consumed by European populations [4, 6], whereas, in ancestral nutrition, fatty acid consumption was only approximately 20–30% of total energy [4, 7, 8]. As a result of the increased consumption of LA-rich vegetable oils associated with the Western diet, n-6 PUFA consumption has become progressively much higher than that of n-3 PUFA [9]. Optimal dietary intakes of the n-6 : n-3 ratio should be around 1–4 : 1. However, according to the nutritional changes described above in the Western diet, this ratio has now increased to be within the range of 10 : 1 to 20 : 1 [4]. In parallel, there are coinciding increases in the incidence of diseases involving inflammatory processes such as cardiovascular disease, obesity, IBD, rheumatoid arthritis, and cancer. Neurodegenerative and psychiatric illnesses such as AD and depression are other examples [10]. A study carried out by Hassan and Hanachi, involving 984 Iranian women, suggested that a good dietary pattern rich in fruits, legumes, vegetables, cereals, and fish, rich in n-3 PUFA, can decrease the likelihood of developing the Metabolic Syndrome [11]. Another study performed in France, involving 912 men, concluded that a low consumption of fish rich in n-3 PUFA is associated with a higher probability of developing the Metabolic Syndrome [12]. Thus, high intake of n-6 PUFA, along with low intakes of n-3 PUFA, shifts the physiological state to one that is proinflammatory and prothrombotic with increases in vasospasm, vasoconstriction, and blood viscosity and the development of diseases associated with these conditions.

PUFA play an important role in the composition of all cell membranes where they maintain homeostasis for correct membrane protein function and influence membrane fluidity, thus regulating cell signalling processes, cellular functions and gene expression [13]. Other functions of PUFA require their metabolism to more highly unsaturated members of their family. For example, LA is converted to AA (20:4n-6) via *γ*-linolenic acid (GLA, 18:3n-6) and dihomo-*γ*-linolenic acid (DGLA, 20:3n-6). By the same set of enzymes, ALA can be converted to EPA (20:5n-3) and DHA (22:6n-3). The primary site for PUFA metabolism is the liver; however, it can also take place in various other tissues [14]. It is these longer chain metabolites of LA and ALA that are of major clinical importance within different organs such as the brain, kidney, and liver [15–17]. Cyclooxygenases (COX) and lipoxygenases (LOX) can convert AA to the 2-series of prostaglandins, the 2-series of thromboxanes, and the 4-series of leukotrienes. These are very important, active and short-lived hormones termed “eicosanoids” which are involved in various pathological processes involving inflammatory conditions such as atherosclerosis, obesity, and IBD [13].

Since PUFA give rise to a variety of biologically active compounds which all have important roles in pathological and physiological processes, a proper understanding is needed regarding the contribution these active compounds have on the coinciding increases in inflammatory diseases seen with the disruption of the balance in the ratio of n-6 : n-3 associated with the Western diet.

## 2. Metabolism of n-6 Fatty Acids and Biosynthesis of Eicosanoids

**Figure 1:**
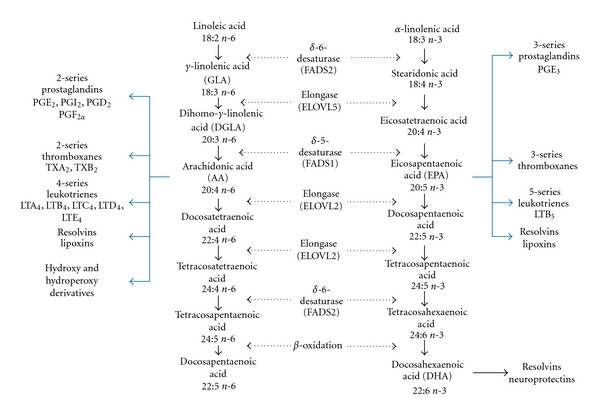
Metabolism of n-6 and n-3 PUFA. The metabolism of PUFA is a complex process involving several enzymes of desaturation, elongation, and *β*-oxidation. Shown here is the pathway of both n-6 and n-3 PUFA metabolism to more unsaturated, long-chain members of each family. Also shown are their respective eicosanoid derivatives. Data elaborated from [21].

Linoleic acid can be metabolized to other more unsaturated, long-chain members of the n-6 family by the insertion of additional double bonds during consecutive elongation and desaturation mechanisms (Figure 1). The initial rate limiting desaturation of LA to GLA is catalysed by the enzyme delta-6-desaturase (FADS2) [19]. Elongation then takes place to convert GLA to DGLA, by elongation of very long-chain fatty acids (ELOVL) 5, and finally a cycle of elongation and desaturation by delta-5-desaturase (FADS1) generates AA [20].

The importance of the FADS2 gene in LC-PUFA synthesis has recently been demonstrated in mice [19, 22]. The first study by Stoffel et al. demonstrates that loss of the FADS2 gene abolishes synthesis of LC-PUFA with further downstream effects on the COX and LOX pathways, eventually leading to hypogonadism and sterility of male and female mice [19]. Further demonstrated by this FADS2 null model was the pivotal role PUFA-substituted phospholipids play in establishing cell polarity, shown here for tight junctions of Sertoli cells of the testis and the gap junction network between ovarian follicle cells [19]. Stroud et al. demonstrated impairment of male reproduction and also both dermal and intestinal ulceration in FADS2 null mice [22].

Elongation of very long-chain fatty acids (ELOVL) 5 is one of seven mammalian fatty acid condensing enzymes involved in microsomal fatty acid elongation [20]. ELOVL5 is required for the elongation of GLA to DGLA. Studies using liver microsomal protein from ELOVL5 null mice found greater tissue accumulation of GLA and a decrease in the levels of downstream metabolism products such as AA for n-6 metabolism and DHA for n-3 metabolism. The metabolic consequence of this reduction of AA and DHA was the activation (or derepression) of sterol regulatory element-binding protein (SREBP)-1c. Activation of this transcription factor (as will be discussed in further detail later) in ELOVL5 null mice resulted in the activation of further genes involved in fatty acid and triglyceride synthesis, which culminated in the development of hepatic steatosis [20].

There are many other factors involved in the regulation of delta-5-desaturase and delta-6-desaturase enzyme activity. For example, decreased activity in both delta-5 and delta-6 desaturases have been demonstrated in the liver of obese NAFLD patients [23]. Glucagon, adrenaline, glucocorticoids, and thryroxin depress delta-5-desaturase and delta-6-desaturase activity [24]. Low delta-6-desaturase enzyme activity was reported in diabetic rats where insulin acts as a well-known delta-6-desaturase stimulator [25]. Since LA and ALA are metabolized by the same set of enzymes, a natural competition exists between these two fatty acids, whereby delta-5-desaturase and delta-6-desaturase will exhibit affinity to metabolize n-3 over n-6 PUFA, provided that they exist in a ratio of 1 : 1–4. However, the higher consumption of LA, as now seen in the Western diet, shows an increase in the preference of these enzymes to metabolize n-6 PUFA, leading to AA synthesis, despite the fact that these enzymes show higher affinity for n-3 PUFA [26]. Supplementation of the diet with EPA and DHA has been shown to correct this imbalance by partially replacing AA from the cell membranes of platelets, erythrocytes, neutrophils, monocytes, and hepatocytes where AA is usually found in high proportions [27].

The intermediates of PUFA metabolism can either be incorporated into phospholipids or undergo further elongation/desaturation steps. In the n-6 pathway, AA, synthesized from the desaturation of DGLA by delta-5-desaturase (FADS1), can be further elongated by ELOVL2 to docosatetraenoic acid (C22:4n-6) or to its respective set of eicosanoids via COX and LOX enzymes. The importance of ELOVL2-derived PUFA in mammals has recently been demonstrated in ELOVL2-ablated mice, thus demonstrating the importance of this elongase enzyme [28]. This study showed the role ELOVL2 plays in the elongation of C20 and C22 PUFA in order to produce C24:4n-6 up to C30:5n-6 PUFA in testis, where they are required for normal spermatogenesis and fertility [28]. Binding of growth factors and hormones to membrane receptors leads to activation of phospholipase A_2_ which releases AA from the cell membrane where the free acid can become a substrate for eicosanoid biosynthesis through the activities of COX and LOX [29]. The eicosanoids derived from AA are synthesized in larger quantities than ever before due to increases in dietary intake [4].

Eicosanoids are biologically active lipids and include prostaglandins (PGs), thromboxanes (TXs), leukotrienes (LTs), and hydroxyeicosatetraenoic acids (HETEs) which have all been implicated in various pathological processes such as inflammation and cancer (Table 2) [30]. When they are present in high quantities, they influence various metabolic activities besides inflammation such as platelet aggregation, haemorrhage, vasoconstriction, and vasodilation [18]. In general, AA-derived eicosanoids are proinflammatory but they have important homeostatic functions in regulating both the promotion and resolution of inflammation in the immune response [31]. In contrast, it is known that the n-3 PUFA and their LC-derivatives mostly promote anti-inflammatory activities [32]. In a recent study involving 250 clinically stable, chronic obstructive pulmonary disease (COPD) patients, higher intakes of n-3 PUFA were associated with lower proinflammatory cytokine concentrations (e.g., tumour necrosis factor alpha (TNF*α*)) while higher n-6 PUFA intake was associated with higher proinflammatory interleukin-6 (IL-6) and C-reactive protein (CRP) concentrations in the diseased state [33]. While COPD is a complex chronic inflammatory condition, it is interesting to see the association between dietary intake of n-6 versus n-3 PUFA on serum inflammatory markers associated with the disease [33]. Despite ample evidence that increased dietary consumption of n-6 PUFA induces a proinflammatory response in the host, it must be reported that recent studies have also shown the opposite [34, 35]. A recent review has suggested that n-6 PUFA have some anti-inflammatory actions such as those of the n-3 PUFA [36]. For example, mean serum CRP concentrations tended to decrease with increased n-6 PUFA consumption in both Japanese men [34] and women [35]. Nevertheless, evidence of these associations is limited.

Metabolism of AA by the COX enzymes (COX-1, a constitutive enzyme, or COX-2, an inducible enzyme) leads to the synthesis of the 2-series of prostaglandins: PGE_2_, PGI_2_, PGD_2_, and PGF_2*α*_ (largely produced by monocytes and macrophages) and thromboxanes A_2_ and B_2_. Collectively, the prostaglandins and thromboxanes are referred to as the prostanoids. The synthesis of AA-derived eicosanoids is, however, dependent on the concentration of DGLA, as DGLA competes with AA for COX and LOX. When DGLA is in excess, it inhibits the synthesis of AA-derived eicosanoids due to its higher affinity for the COX and LOX enzymes [40]. The activity of 5-LOX metabolises AA to hydroxyl and hydroperoxy derivatives: 5-HETE and 5-hydro-peroxyeicosatetraenoic acid (5-HPETE). These derivatives in turn produce the 4-series of leukotrienes: leukotriene A_4_ (LTA_4_), leukotriene B_4_ (LTB_4_), leukotriene C_4_ (LTC_4_), leukotriene D_4_ (LTD_4_), and leukotriene E_4_ (LTE_4_). Monocytes, macrophages, and neutrophils produce LTB_4_, while mast cells, eosinophils and basophils produce LTC_4_, LTD_4_, and LTE_4_ [41].

Prostaglandin overproduction has various proinflammatory effects. For example, PGI_2_ and PGE_2_ exert their acute inflammatory response in arthritis [42, 43]. PGE_2_ can also increase its own synthesis through induction of COX-2 leading to the production of the proinflammatory cytokine IL-6 in macrophages [41, 44]. TXB_2_ is a potent vasoconstrictor and platelet activator. LTB_4_ has many proinflammatory functions, acting as an important activator of neutrophils, a chemotactic agent for leukocytes, induces release of lysosomal enzymes, accelerates reactive oxygen species (ROS) production, and increases vascular permeability [21]. LTB_4_ also leads to the production of inflammatory cytokines like TNF-*α*, interleukin 1 beta (IL-1*β*) and IL-6 by macrophages [45]. However, the overall pathophysiological outcome will depend on the cells present, the nature of the stimulus, the timing of eicosanoid generation, the concentrations of different eicosanoids generated, and the sensitivity of target cells and tissues to the eicosanoids generated [1].

In contrast, EPA can also act as a substrate for COX and LOX enzymes and gives rise to an entirely different set of eicosanoids (Table 2). These are the 3-series prostaglandins and thromboxanes and the 5-series leukotrienes, which are considered to be less inflammatory or even anti-inflammatory in comparison to the eicosanoid family derived from AA [46].

## 3. How n-6 PUFA-Derived Eicosanoids Influence Inflammatory Responses

The mode by which prostaglandins and leukotrienes exert their biological homeostatic and inflammatory actions depends on binding to their respective G-protein coupled receptors (GPCRs). Specific GPCRs have been identified for all the prostanoids, where there are at least nine known prostanoid receptor forms in mouse and man [47, 48]. Although most of the prostaglandin GPCRs are localised at the plasma membrane of platelets, vascular smooth muscle cells, and mast cells, some are situated at the nuclear envelope [49]. Four of these receptor subtypes bind PGE_2_ (EP_1_–EP_4_), two bind PGD_2_ (DP_1_ and DP_2_), and more specific receptors bind PGF_2*α*_, PGI_2_, and TXA_2_ (FP, IP, and TP, resp.) [37]. PGE_2_ and PGI_2_ are the predominant proinflammatory prostanoids, and, through their activation of EP2 and IP, respectively, they can increase vascular permeability and leukocyte infiltration. In individuals with asthma, a bronchial allergen leads to enhanced PGD_2_ production. Thus, during asthmatic attacks in humans, PGD_2_ is released in large amounts by mast cells [50]. PGD_2_ can also promote inflammation via DP_2_ through activation of eosinophils [47, 51].

Four distinct GPCRs for leukotrienes have been characterized. LTB_4_ interacts with BTL_1_ and BTL_2_ through which important roles in host defence of cells and inflammation are mediated [52]. LTB_4_ induces leukocyte infiltration and as already mentioned above leads to the release of proinflammatory cytokines. As an example, in patients with IBD, the colonic mucosa contains 3- to 7-fold higher counts of cells expressing the 5-LOX pathway, thus increasing the tissue synthesis of LTB_4_ [53]. LTC_4_ and LTD_4_ can contract smooth muscle by interacting with two subtypes of cysteinyl leukotriene receptors, CysLT_1_ and CysLT_2_ [54].

The proinflammatory effects of the AA-derived prostanoids and leukotrienes have been described [37]. A mechanism has been proposed whereby a coordinated program for resolution initiates in the first few hours after the inflammatory response. A switch occurs whereby the AA-derived prostanoids and leukotrienes, which have set the inflammatory response to begin, undergo further metabolism to become another generation of eicosanoids derived from AA termed lipoxins and hence terminate inflammation at the local contained sites [55]. Since these lipoxins are involved in the resolution of the acute inflammation that occurs as a result of the overproduction of the proinflammatory eicosanoids derived from AA, they are said to have “pro-resolving” and anti-inflammatory functions. These events coincide with the biosynthesis of resolvins and protectins from n-3 fatty acids, which act to shorten the period of neutrophil infiltration [55]. However, while the initial response of the AA-derived eicosanoids to promote inflammation is beneficial in one respect, for example, in the control of blood flow and vessel dilation, the increase in the ratio of n-6 : n-3 PUFA leads to an overall increase in the production of proinflammatory cytokines and an unnecessary over reactive inflammatory response leading to the pathogenesis of inflammatory diseases. In addition, the decrease in consumption of n-3 PUFA which leads to an overall decrease in resolvin and protectin production is detrimental to the inflammatory response as these products, which have the ability to dominate the resolution phase of inflammation, can no longer exert this potential; thus, the inflammatory response cannot be terminated effectively.

## 4. n-6 PUFA Regulation of Inflammatory Gene Expression

Nuclear receptors are a family of ligand-activated transcription factors that either directly or indirectly control various genes of lipid metabolism and inflammatory signalling [21]. Upon ligand binding, nuclear receptors can undergo conformational changes which dissociate corepressors and facilitate recruitment of coactivator proteins to enable transcription activation [21, 56]. LC-PUFA and their eicosanoid derivatives can act as ligands for these transcription factors and hence elicit changes in gene expression by governing the activity of nuclear transcription factors. The regulation of gene expression by dietary fats is believed to be one of the greatest factors impacting on the development of certain diseases of affluence related to the Metabolic Syndrome, such as hepatic steatosis and NAFLD.

The peroxisome proliferator-activated receptor (PPAR) family is composed of three proteins: PPAR*α*, PPAR*β*/*δ*, and PPAR*γ*, and, although they each have different tissue distributions, their biological functions overlap [57]. The PPARs have emerged as important regulators of metabolic and inflammatory signalling, in both metabolic disease and immunity [57]. The role PPAR*α* plays in the regulation of genes involved in lipid metabolism was first identified in the early 1990s, on the basis of being a target of the hypolipidaemic fibrate drugs and other compounds that induce peroxisome proliferation in rodents [57, 58]. PUFA, especially those of the n-3 family and their eicosanoid derivatives, are ligands for the PPARs. The n-3 fatty acids EPA and DHA have been shown to be more potent as *in vivo* activators of PPAR*α* than the n-6 fatty acids [59–62]. Once PPARs become activated, they form heterodimers with the retinoid X receptor (RXR) and these dimers then bind to PPAR responsive elements (PPREs) in target genes to alter coactivator/corepressor dynamics and induce transcription [63]. PPAR*α* has recently been shown to exert hypolipidaemic effects through activation of skeletal muscle, cardiac and hepatic genes encoding proteins which are involved in lipid oxidation [63–65]. Thus, the PPARs, particularly PPAR*α*, play an important role in insulin sensitization, atherosclerosis, and metabolic diseases. In the regulation of inflammatory signalling, PPARs inhibit nuclear factor-kappa B (NF*κ*B) expression.

NF*κ*B, another transcription factor regulated by PUFA, is found in almost all animal cell types, has a crucial role in inflammatory signalling pathways, and plays a key role in regulating the immune response to infection. It controls several cytokines (e.g., IL-1, IL-2, IL-6, IL-12, and TNF-*α*), chemokines (e.g., IL-8, monocyte chemoattractant protein-1), adhesion molecules, and inducible effector enzymes (e.g., inducible nitric oxide synthase and COX-2) [2]. NF*κ*B becomes activated as a result of a signalling cascade triggered by extracellular inflammatory stimuli (such as free radicals, bacterial or viral antigens), which involves phosphorylation of an inhibitory subunit of NF*κ*B (I*κ*B), which in turn allows the translocation of the remaining NF*κ*B dimer to the nucleus, with the result of an increase in expression of inflammatory genes [66]. Since the n-3 LC-PUFA show anti-inflammatory action, they inhibit NF*κ*B activity. As an example, both EPA and DHA have been shown to block the activity of NF*κ*B through decreased degradation of I*κ*B, in human monocytes and human THP-1 monocyte-derived macrophages [67, 68]. However, this effect is not observed to the same extent with n-6 LC-PUFA, due to potency in the inhibition of NF*κ*B [69]. Interestingly, 5-LOX, the enzyme which converts AA to the 4-series leukotrienes and 5-HETE, translocates into the nucleus in association with NF*κ*B [70, 71].

SREBP-1c is a transcription factor required for the insulin-mediated induction of hepatic fatty acid and triglyceride synthesis. Responsive targets in mammalian cells include genes of fatty acid metabolism, such as fatty acid synthase (FAS), and its expression is most commonly found in high levels in macrophages, liver, white adipose tissue, adrenal glands, and the brain of both mice and humans [72]. PUFA have the ability to modulate SREBP-1c activity and expression. For example, n-3 LC-PUFA have been shown to suppress SREBP-1c gene expression and so inhibit transcription of lipogenic and hepatic genes involved in lipid biosynthesis [73, 74]. Studies have shown that a decrease in hepatic SREBP-1c leads to a decrease in hepatic FAS, thus lowering lipid accumulation within the liver [75–77]. However, the n-3 PUFA are more potent inhibitors of SREBP-1c, than the n-6 PUFA [21], and this will be discussed in more detail further on. More recently, the liver X receptors (LXR-*α* and -*β*) have been shown to play a major role in lipogenesis through regulation of transcription of the gene encoding SREBP-1c [77]. This study concluded that the downregulation of SREBP-1c transcription by n-3 PUFA results from attenuated transactivation of the ligand-activated nuclear receptor LXR-*α* [77]. A more recent study in mice fed an n-3 PUFA depleted diet showed increased activation of SREBP-1c and related pathways which was consistent with increased LXR activity, thus highlighting the importance of n-3 PUFA depletion related to lipid accumulation in the liver [78]. However, in another study by Pawar et al., fish oil fed rats showed a suppression of hepatic SREBP-1c target genes, but no change in expression of genes directly regulated by LXR [79]. Inhibition of LXR may also be an indirect effect of PUFA stimulation of PPAR transcription factors [21]. Cross-talk between PPAR*α* and LXR via SREBP-1c has been reported, whereby overexpression of PPAR*α* inhibited LXR-induced SREBP-1c promoter activity, through a reduction of LXR binding to its activator, RXR [80]. Both n-6 and n-3 PUFA are often interchangeable in regulating gene expression. However, it is well known that n-3 PUFA are more potent ligands to these nuclear receptors than n-6 PUFA [21]. Through n-3 PUFA-mediated activation of PPAR*α* and inhibition of SREBP-1c, lipid biosynthesis can be reduced and lipid degradation can be increased [21, 29].

By targeting the transcription of various nuclear receptors involved in regulating lipogenic gene expression through dietary fatty acids, prevention of certain diseases related to the Metabolic Syndrome, such as hepatic steatosis and NAFLD, can be reduced in the future. The contribution n-6 PUFA make to the development of liver disease due to the increased consumption of LA-rich foods and the decreased consumption of ALA rich foods is phenomenal and will be discussed in further detail. Already discussed are the positive contributions of n-3 PUFA in the prevention of lipid biosynthesis in various organs, such as the liver, for example, through the activation of PPAR*α* and inhibition of NF*κ*B and SREBP-1c. However, since these n-3 PUFA are more potent ligands for these nuclear receptors and Western diets overall consumption of n-6 : n-3 has increased dramatically over the last 50 years in particular, what now becomes the fate of the these nuclear receptors and how have our dietary changes impacted upon our health status through regulation of inflammatory gene expression? More importantly, determination of the molecular and cellular mechanisms regulated by PUFA may help identify novel sites for pharmacological intervention.

## 5. n-6 PUFA Contribution to Chronic Inflammatory Conditions in Humans

**Figure 2:**
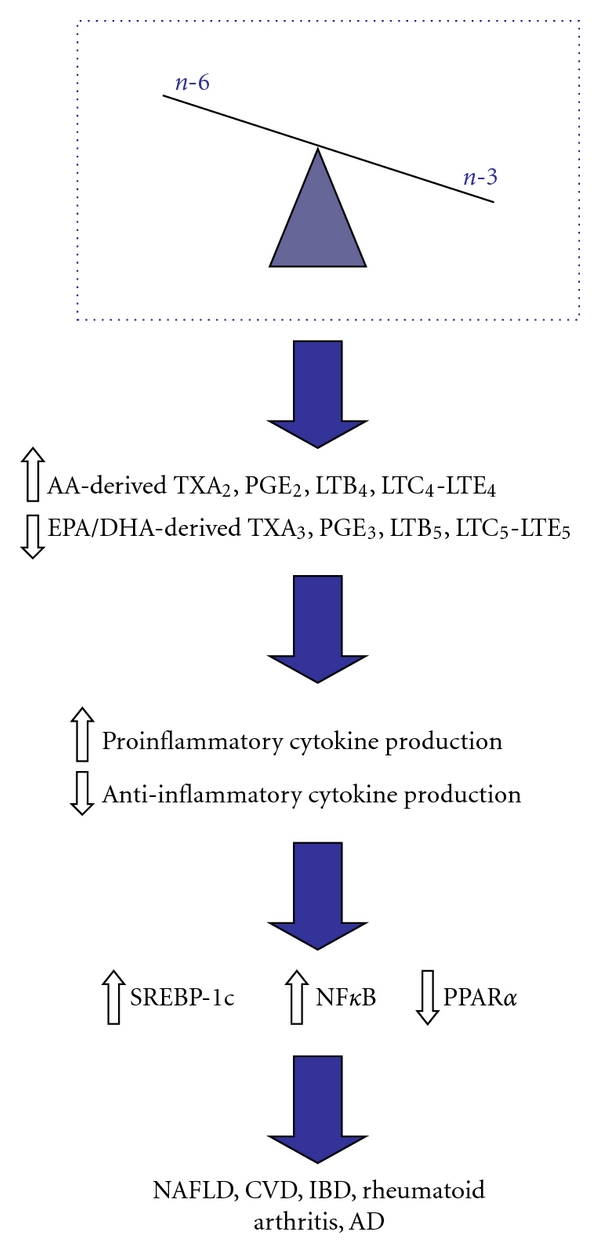
Effects of unbalanced n-6 : n-3 dietary fatty acid intake on development of various diseases of inflammation. Dietary imbalance in the consumption of n-6 and n-3 PUFA, representative of the Western diet. Greater consumption of n-6 PUFA leads to an increase in their metabolism to their LC-PUFA derivatives (AA). Decreases in n-3 PUFA consumption leads to a decrease in their metabolism to their LC-PUFA derivatives (EPA/DHA). The increase in AA in cell membrane phospholipids leads to an increase in COX and LOX enzyme production of AA-derived eicosanoids and a decrease in EPA/DHA-derived eicosanoids, leading to an increase in inflammation and proinflammatory cytokine production. This in turn leads to a decrease in PPAR*α* gene expression, while there is an increase in both SREBP-1c and NF*κ*B gene expression. This change in gene expression can also cause an increase in lipogenesis, as well as increasing inflammation. The result is an increase in various diseases of inflammation, some of which are highlighted in the figure.

Clinical studies indicate that inflammation is at the base of many diseases including NAFLD, cardiovascular disease, atherosclerosis, IBD, and neurodegenerative diseases such as AD (Figure 2). The contribution of n-6 PUFA to these inflammatory conditions will be reviewed below with a particular focus on NAFLD.

### 5.1. Nonalcoholic Fatty Liver Disease (NAFLD) 

NAFLD is often described as the hepatic component of the Metabolic Syndrome and is rapidly becoming a serious public health problem [81]. The range of liver damage associated with NAFLD begins with steatosis and can often persist to further steatohepatitis (NASH), advanced fibrosis and cirrhosis [82]. NAFLD itself is an independent risk factor for cardiovascular disease (CVD). Occurrence of NAFLD is much higher in subgroups of the population with obesity, Metabolic Syndrome, and type 2 diabetes, whereby prevalence in developing the disease for those with type 2 diabetes may be as high as 70% [82, 83]. Both nutritional factors and alterations in lipid metabolism of the liver are the primary metabolic abnormalities which lead to hepatic steatosis [84].

The role of n-3 LC-PUFA as a potential therapeutic target in the pathogenesis of NAFLD has recently been demonstrated [85]. Within the liver, n-3 LC-PUFA presence is associated with an increased ability to direct fatty acids away from triacylglycerol storage and to enhance their oxidation. However, n-3 LC-PUFA levels are decreased in the hepatic tissue of patients with NAFLD [86, 87]. Depletion of n-3 LC-PUFA within the livers of NAFLD patients is a major problem as liver fatty acids now become directed away from oxidation and secretion and instead towards triacylglycerol storage. In addition, a higher n-6 : n-3 LC-PUFA ratio within the liver of NAFLD patients may contribute to the development of fatty liver due to a derangement in the capacity to regulate liver lipid metabolism [86]. A recent comparative review also demonstrated various mechanisms through which consumption of fish oil has been beneficial in the alleviation of NAFLD such as (i) decreased plasma nonesterified fatty acids (NEFA) concentrations; (ii) decreased *de novo* lipogenesis, very low-density lipoprotein (VLDL) export, and plasma triglyceride concentrations; (iii) decreased adipocyte size and visceral fat content [88]. The mechanisms which lead to the development of fatty liver, such as impaired fatty acid oxidation and increased *de novo* fatty acid synthesis, are regulated by hepatic gene transcription.

n-3 LC-PUFA regulate lipid metabolism in the liver by acting as ligand activators of the transcription factor PPAR*α*. Activation of PPAR*α* results in the upregulation of genes which are involved in fatty acid and lipid metabolism and which stimulate fatty acid oxidation [17, 89]. In two separate studies employing murine models of NASH, administration of a PPAR*α* agonist prevented steatohepatitis and reversed the established disease [90, 91].

VLDL is a type of lipoprotein made by the liver from triglycerides, cholesterols, and apolipoproteins. Within the bloodstream, VLDL transports cholesterol from the liver, thus enabling fats to move within the bloodstream, and it is here that VLDL itself also acts as a precursor to low-density lipoprotein (LDL), often referred to as “bad cholesterol.” PPAR*α* activation increases the secretion of apoliopoprotein B-100 (apo B-100), which is the main structural protein of VLDL, and upregulates the expression of liver fatty acid binding protein (LFABP) which is essential for the secretion of apo B-100 [92, 93]. Since n-3 LC-PUFA upregulate PPAR*α*, hepatic fatty acid oxidation has the potential to occur within the liver, and, since more apo B-100 is secreted out of the liver, less VLDL is synthesized, with the result of less of this harmful cholesterol entering the bloodstream, where the downstream further effects on the development of atherosclerosis are attenuated [94]. However, with the reduced availability of n-3 LC-PUFA from dietary intake and the increases in n-6 PUFA consumption, PPAR*α* does not become activated to its full potential. This results in PUFA favouring fatty acid and triglyceride synthesis over fatty acid degradation. As demonstrated by PPAR*α* ^−/−^ mice, rates in their ability to oxidise fatty acids are decreased during periods of food deprivation; thus, they develop characteristics of adult-onset diabetes including fatty livers, elevated blood triglyceride concentrations, and hyperglycemia [95].

n-3 LC-PUFA are also involved in the negative regulation of the transcription factor SREBP-1c within the liver, thus acting as inhibitors in the expression of lipogenic genes such as FAS [73]. The effect n-3 LC-PUFA have on SREBP-1c is to reduce endogenous lipid production and accumulation of triglycerides in the liver [96], and this is achieved by reducing the amount of mature SREBP-1c available for *de novo* lipogenesis within the nucleus [97]. Therefore, depletion of n-3 LC-PUFA and an increase in the ratio of n-6 : n-3 LC-PUFA in the liver of NAFLD patients results in fatty acid and triacylglycerol synthesis over oxidation, again leading to fatty liver. A recent study by Pachikian et al. using mice fed a depleted n-3 PUFA diet showed increases in hepatic activation of SREBP-1c leading to increased lipogenesis, contributing to hepatic steatosis [78]. This is consistent with a previous study in rats fed an n-3 PUFA-depleted diet whereby hepatic accumulation of triglycerides and esterified cholesterol led to both macro-and microvesicular steatosis caused by changes in the fatty acid pattern that resulted from n-3 PUFA depletion [98].

Another mechanism involved in the depletion of n-3 LC-PUFA from the liver of obese NAFLD patients and which further exacerbates the disease progression is the decreased liver fatty acid delta-5 and delta-6 activity in these patients [23]. Impairment of these enzymes affects the desaturation and elongation pathways of LA and ALA, which are required for the synthesis of their LC-PUFA derivatives within the liver [99]. Decreased activity in both delta-5 and delta-6 desaturases has been demonstrated in the liver of obese NAFLD patients [23]. This may be attributed to the lower intake of ALA (n-3 precursor), the imbalance in the n-6 : n-3 LC-PUFA ratio which occurs in the liver and higher consumption of trans isomers (18 : 1, n-9 trans) inhibiting delta-6 desaturase [86]. The depletion of n-3 LC-PUFA within the liver of these patients resulting from the decrease in delta-5 and delta-6 desaturase activity may lead to further development of steatosis by altering the activity of PPAR*α* and SREBP-1c [23]. This will determine a metabolic imbalance favouring lipogenesis over fatty acid oxidation since n-3 LC-PUFA depletion induces SREBP-1c expression and upregulation of lipogenic genes [84]. In general, it is also understood that the adipose tissue acts as a suitable biomarker for dietary fatty acid intake. Considering that in NAFLD, there is an enhancement in n-6 adipose tissue content and a significant decrease in n-3 adipose tissue content, this suggests that while there is an adequate amount of n-6 fatty acids for metabolism within the liver, n-3 fatty acids cannot be metabolized to the same extent due to inadequate dietary intake. Also, decreased dietary intake of n-3 PUFA constitutes a limiting factor for the production of n-3 LC-PUFA in liver lipids of NAFLD patients, resulting from the competition between the two metabolic pathways (Figure 1), particularly at the desaturation steps [86]. Thus, a dietary imbalance comprising inadequate intake of n-3 PUFA and an excess intake of n-6 PUFA leads to defective desaturation of PUFA [86].

Oxidative stress caused by the accumulation of liver triglycerides and insulin resistance are major contributors in the pathogenesis of NAFLD [100]. Both oxidative stress and mitochondrial dysfunction are often associated with the increased production of ROS and proinflammatory cytokines related to NAFLD [82]. Recent human studies have described a strong association between the severity of NASH and the degree of oxidative stress [100–102]. The increased prooxidant activity associated with oxidative stress leads to elevation in hepatic lipid peroxidation status. Lipid peroxidation can also cause immunological dysfunction, which could lead to the development of hepatic fibrogenesis [82]. This could potentially lead to an increase in the release of 4-hydroxy-20-nonenal (HNE), which can bind hepatocyte proteins forming new antigens and therefore provoking a harmful immunological response [82]. For example, Seki et al. reported a correlation between hepatic expression of HNE and the degree of severity of necroinflammation and fibrosis [103]. Oxidative stress associated with NAFLD has also been shown to increase production of proinflammatory cytokines. This hepatotoxicity associated with the production of inflammatory cytokines induced through oxidative stress may indirectly activate transcription factors such as NF*κ*B. The accumulation of NEFAs within hepatocytes of NAFLD patients is another source of NF*κ*B activation [104].

Oxidative stress and changes in dietary intake trends may contribute to low hepatic LC-PUFA [105]. The increase in lipid peroxidation associated with NAFLD, as discussed, may contribute to the decrease in LC-PUFA, as they are particularly susceptible to lipid peroxidation [105, 106]. Thus, oxidative stress-dependent lipid peroxidation may represent an alternative mechanism to liver n-3 LC-PUFA depletion in NAFLD [99]; since PUFA are more susceptible to peroxidation and the greater availability of n-6 LC-PUFA in the livers of NAFLD patients results in enhanced peroxidation of these LA derived LC-PUFA into their eicosanoid derivatives [99]. For example, LTB_4_, an AA-derived eicosanoid, is involved in acceleration of ROS production. The increased production of proinflammatory cytokines and eicosanoids, produced from n-6 PUFA metabolism, cause enhanced liver Kupffer cell production of inflammatory cytokines causing activation of NF*κ*B, further exacerbating systemic and hepatic insulin resistance with worsening inflammation and fibrosis [82]. Insulin resistance as seen in NAFLD may be related to the depletion in n-3 LC-PUFA because they are expected to modify membrane-mediated processes such as insulin signalling [107].

In summary, the depletion of n-3 LC-PUFA, the decrease in the ratio of product/precursors of LC-PUFA, the increase in n-6 PUFA, and the increase in n-6 LC-PUFA derived eicosanoid production within the liver all contribute to the development of NAFLD and related pathophysiologies such as insulin resistance. Recently, the relationship between the n-6 : n-3 PUFA ratio within the liver and severity of steatosis was demonstrated [108]. In this study, patients with NAFLD showed significant correlation between the n-6 : n-3 PUFA ratio and the quantity of hepatic triglycerides, as a marker of the severity of hepatic steatosis [108]. Defective desaturation of PUFA due to inadequate intake of n-3 PUFA, and a higher intake of n-6 PUFA further enhances the contribution of desaturase inhibition in NAFLD.

### 5.2. Other Inflammatory Conditions Involving n-6 PUFA

#### 5.2.1. Cardiovascular Disease and Atherosclerosis

Atherosclerosis is now considered a “systemic disease” characterised by low-grade arterial inflammatory lesions that can mature along with disease progression [109]. It is the underlying cause of coronary heart disease (CHD), and abnormalities in the metabolism of essential fatty acids that are characteristic of the associated risk factors [110]. Under normal physiological conditions, healthy endothelial cells synthesise and release adequate amounts of NO, PGI_2_, and PGE_1_, maintaining a downstream balance between pro- and anti-inflammatory molecules. However, in atherosclerosis, this balance becomes disrupted, leaning towards an increase in the production of proinflammatory cytokines such as IL-1, IL-2, IL-6 and TNF-*α*, resulting in the further progression of the disease [110]. These proinflammatory cytokines can induce oxidative stress by enhancing the production of ROS by monocytes, macrophages, and leukocytes. Since PUFA and their eicosanoid derivatives modulate inflammation, they play a significant role in this disease [26]. Decreases in ALA-derived LC-PUFA such as EPA and DHA seen in endothelial cell PUFA deficiency, increases the production of proinflammatory cytokines and free radicals which results in the development of insulin resistance [26]. As an example, early studies in Greenland Eskimos, a population consuming a high-fat diet, but rich in n-3 PUFA, showed that ingestion of EPA and DHA led to decreases in the mortality rate from CVD [111]. Similarly, Japanese populations eat more fish than North Americans and present a lower rate of acute myocardial infarction and atherosclerosis [112, 113]. Other later studies have further demonstrated strong associations between n-3 PUFA intake and decreased risks of CVD [114–116].

The role of n-6 PUFA in CVD is much more complex than the role of n-3 PUFA. PGE_2_, PGF_2*α*_, TXA_2_, and LTs produced from AA metabolism are proinflammatory [110]. TXA_2_ acts as a potent vasoconstrictor and powerful activator of platelet aggregation [117]. Studies have shown that TXA_2_ promotes the initiation and progression of atherosclerosis by regulating platelet activation and leukocyte-endothelial cell interactions [118]. LTB_4_ acts as a potent chemotactic agent, inducing the generation of ROS, activating neutrophils, and inducing the aggregation and adhesion of leukocytes to the vascular endothelium [110]. The leukotrienes LTC_4_, LTD_4_, and LTE_4_ induce vasoconstriction and bronchospasm [110].

Since AA is derived from LA, a reduction of LA intakes will reduce tissue AA content, which in turn will reduce any inflammatory potential and therefore lower the risk for CVD [119]. There are many other lines of evidence that link LA with atherosclerosis. Endothelial dysfunction (ED) is a characteristic of early-state atherosclerosis common in patients with insulin resistance and diabetes [120]. A recent review by Simopoulos reported that diets enriched in LA increase the LA content of LDL and its susceptibility to oxidation, whereby oxidative modification increases the atherogenicity of LDL cholesterol [121]. Studies have also shown that in patients with type 2 diabetes susceptible of developing ED, there are substantial increases in LA concentrations in all LDL subfractions [122]. Cellular oxidative stress associated with LA oxidation of LDL and LA mediated ED is a critical signal transduction pathway involved in NF*κ*B activation, whereby NF*κ*B is critical for the expression of inflammatory genes associated with ED [120]. The susceptibility of LDL to oxidation by LA and its associated metabolites is linked to the severity of coronary atherosclerosis development [121, 123]. Despite the evidence to suggest that n-6 PUFA consumption increases the risk of developing CVD, recent evidence has suggested that both LA and ALA have the ability to prevent CVD [34]. In this study, LA significantly reduced levels of CRP, an inflammatory marker, upregulated in CVD in Japanese men [34]. However, other evidence to suggest that n-6 PUFA have an anti-inflammatory effect when consumed in such high quantities, such as that seen in the Western diet, is limited.

Since it has been proposed that diets high in LA reduce ALA metabolism [124] and since ALA metabolites such as EPA/DHA have been shown to reduce mortality rates from CVD [111–113], the balance of n-6 to n-3 PUFA is important in the prevention of atherosclerosis and CVD.

#### 5.2.2. Inflammatory Bowel Disease

IBD is classified as a group of chronic systemically natured diseases of unclear pathology which cause inflammation of the digestive tract, including Crohn's disease (CD) and ulcerative colitis (UC) [125]. While environmental factors indeed play a significant role in the etiology of the disease, more recent attention has been placed on various dietary and nutritional factors, specifically the lipid components of the diet as triggers of IBD [125, 126]. It is difficult to suggest that dietary influences or supplementation can reduce the incidence of IBD or impact beneficially (through anti-inflammatory effects) upon the disease progression since, like many chronic diseases, IBD is multifactorial. Despite this, lower prevalence of IBD has been observed with consumption of diets rich in n-3 LC-PUFA derived from fish oils, such as that seen of the Greenland Eskimos [127, 128]. It has also been reported that patients of IBD who supplement their diets with n-3 PUFA show anti-inflammatory actions, with decreased production of LTB_4_ by neutrophils and colonic mucosa, resulting from incorporation of the n-3 PUFA into the gut mucosal tissue [129, 130]. A recent study using IL-10 knockout mice (mice that spontaneously develop colitis) demonstrated significantly reduced colonic inflammation when fed n-3 PUFA-rich fish oil as compared with mice that were fed n-6 PUFA-rich corn oil [131]. In Japan, increased reports in the incidence of IBD correlate with the increased dietary intake of n-6 PUFA [132, 133]. Importantly, while n-3 PUFA show decreased production of LTB_4_ by neutrophils and colonic mucosa [129, 130], metabolism of AA increases the production of LTB_4_ within the inflamed intestinal mucosa of IBD [134]. A more recent report demonstrated abnormal prevalence of the enzymes that coordinate to generate LTB_4_ from membrane-derived AA in active IBD biopsies [53]. The recruitment of neutrophils and other leukocytes to the IBD gut mucosa seen with colonic injury may be a direct result of the increased ability to generate LTB_4_ from AA [53]. It is clear from the literature that n-3 PUFA have a positive effect on reducing the risk of IBD [135–137]. The situation is less clear for n-6 PUFA, although the proinflammatory eicosanoids derived from AA have been shown to play a crucial role in the pathogenesis of all these related inflammatory disorders. As n-3 PUFA have been shown to alleviate the progression of IBD, while n-6 PUFA have been implicated in the origin of IBD, the importance of a balance in the ratio of n-6 : n-3 PUFA in today's dietary regime is highlighted.

#### 5.2.3. Rheumatoid Arthritis

Rheumatoid arthritis is a long-term disease that leads to inflammation of the joints and surrounding tissues, causing pain, swelling, and impaired function. It is characterised by infiltration of T-lymphocytes, macrophages, and plasma cells into the synovium, with the initiation of a chronic inflammatory state that involves the overproduction of proinflammatory cytokines [138]. Studies have shown that AA-derived eicosanoids, PGE_2_, and PGI_2_ play a role in the pathogenesis of rheumatoid arthritis [42, 139]. PGI receptor-deficient (IP^−/−^) mice subjected to collagen-induced arthritis (CIA) showed a significant reduction in arthritic scores and reduction in IL-1*β* and IL-6 levels in the arthritic paws [139]. Inhibition of both PGE receptors (EP2 and EP4) suppressed inflammatory events and arthritis in CIA. These results suggest that both PGE_2_ and PGI_2_ participate in rheumatoid arthritis. Supplementation with n-3 PUFA has been demonstrated to modulate the activity of inflammatory factors that cause cartilage destruction during arthritis [138, 140]. Moreover, decreasing n-6 PUFA intake (especially AA) down to less than 90 mg/day through an anti-inflammatory lactovegetarian (versus normal Western) diet was shown to improve the clinical symptoms associated with rheumatoid arthritis [141].

#### 5.2.4. Alzheimer's Disease

AD is the most common form of dementia in the elderly, clinically characterised by memory dysfunction, loss of lexical access, spatial and temporal disorientation, and impaired judgement [10]. The pathogenesis of AD is extremely complex, with genetic factors, education, and lifestyle all playing crucial roles in disease onset. However, a poor understanding of the pathogenesis of AD means that there are no curative treatments yet available. Recently, much interest has been shown in the role of diet in both the pathogenesis and prevention of this disease. The role of n-6 PUFA and oxidised eicosanoid derivatives of n-6 PUFA have recently been reviewed as contributing to *β*-amyloid deposition, a hallmark of AD onset and progression [142, 143]. AA is distributed in several different cell types in both the grey and white matter in the brain [10]. The role AA plays in oxidative stress and lipid peroxidation has already been discussed in relation to NAFLD; however, oxidative stress and production of ROS has also been suggested to play a role in AD, thus suggesting a role of AA and lipid oxidation products (eicosanoids) in the onset and progression of the disease [144, 145]. Furthermore, the enhanced consumption of n-6 PUFA leads to an excessive production of the proinflammatory cytokines derived from AA through COX and LOX enzymatic activity which lead to brain damage [146, 147]. As an example, a study using transgenic mice with memory impairment and *β*-amyloid deposition, fed a diet poor in n-3 PUFA but rich in n-6 PUFA, showed that they were found to have a significant decrease in the postsynaptic receptor complex in the brain which regulates memory and learning and a net potentiation of programmed cell death [148]. In contrast, n-3 PUFA may play a role in the prevention of AD. Studies have shown that DHA provides support to learning and memory events in animal models of AD and protection against the disease [149–151]. Another recent epidemiological study indicated a relationship between higher fish consumption and improved cognitive function in later life [152]. Both DHA and EPA have been shown to competitively counteract the production of proinflammatory eicosanoids derived from n-6 PUFA in the brain of AD patients [153]. The neuroprotective role of EPA has been demonstrated since EPA competes with AA for incorporation into cell membrane phospholipids and for oxidation by the COX enzyme, thus exerting anti-inflammatory actions. The resulting production of anti-inflammatory PGE_3_ might result in decreased levels of proinflammatory PGE_2_ [154]. The balance between the n-6 : n-3 PUFA ratio may therefore play a crucial role in the onset of AD. A recent study showed that a lower n-6 : n-3 PUFA ratio was associated with a lower incidence of dementia, especially in depressed patients [155]. Furthermore, we have previously demonstrated in patients with major depression, increases in plasma AA and IL-6 associated with inflammation [156]. Therefore, a dietary pattern consisting of lower n-6 PUFA and higher n-3 PUFA or a more balanced n-6 : n-3 PUFA ratio may be therapeutic in the pathogenesis of AD.

## 6. Conclusion

Increases in the ratio of n-6 : n-3 PUFA, characteristic of the Western diet, could potentiate inflammatory processes and consequently predispose to or exacerbate many inflammatory diseases. The change in ratio and increase in n-6 PUFA consumption change the production of important mediators and regulators of inflammation and immune responses towards a proinflammatory profile. Chronic conditions such as CVD, diabetes, obesity, rheumatoid arthritis, and IBD are all associated with increased production of PGE_2_, LTB_4_, TXA_2_, IL-1*β*, IL-6, and TNF-*α*, whereby the production of these factors increases with increased dietary intake of n-6 PUFA and decreased dietary intake of n-3 PUFA. In conclusion, the unbalanced dietary consumption of n-6 : n-3 PUFA is detrimental to human health, and so the impact of dietary supplementation with n-3 PUFA upon the alleviation of inflammatory diseases, more specifically, NAFLD needs to be more thoroughly investigated.

## Figures and Tables

**Table 1:** PUFA content of dietary components.

Fat type	LA	ALA	AA	EPA + DHA
*Saturated*				
Lard	8600	1000	1070	
Butter fat	2300	1400		
Coconut oil	1400			
Beef tallow	80			
*Unsaturated*				
(1) Monounsaturated				
Peanut oil	23900			
Pecans	20600	1000		
Almonds	9860	260		
Olive oil	8000	950		
Avocado	1970			
(2) Polyunsaturated				
Omega-6				
Safflower oil	74000	470		
Sunflower oil	60200	500		
Soybean oil	53400	7600		
Corn oil	50000	900		
Cotton seed oil	47800	1000		
Walnut	34100	6800	590	
Brazil nut	24900			
Omega-3				
Linseed oil	13400	55300		
Canola oil	19100	8600		
Salmon	440	550	300	1200
Tuna	260	270	280	400
Herring	150	62	37	1700
Trout	74		30	500
Cod	4	2	3	300

Data are expressed as mg/100 g edible portion. Data are elaborated from [13, 14, 18].

Content of fatty acids may vary slightly according to species, sources, and analytical factors.

**Table 2:** Proinflammatory effects of n-6 fatty-acid-derived eicosanoids and anti-inflammatory effects of the n-3 fatty-acid-derived eicosanoids.

Proinflammatory effects of the n-6 fatty-acid-derived eicosanoids			

	Arachidonic acid (n-6) derived eicosanoids	Physiological effects	Organs or cells

Prostaglandins	PGD_2_	Bronchoconstriction Proinflammatory	Bronchi Activation of eosinophils
	PGE_2_	Proarrhythmic Induces fever Causes pain Increases production of IL-6	Vessels Nociceptor sensory neurons
	PGF_2_	Bronchoconstriction	Bronchi
	PGI_2_	Proarrhythmic	Vessels
		Causes pain	Nociceptor sensory neurons

Thromboxanes	TXA_2_	Proaggregation Vasoconstriction Bronchoconstriction	Platelets Vessels Bronchi
	TXB_2_	Proaggregation Vasoconstriction Bronchoconstriction	Platelets Vessels Bronchi

Leukotrienes	LTA_4_		
	LTB_4_	Proinflammatory Chemotaxis Release of reactive oxygen species	Leukocytes Leukocytes Granulocytes
	LTC_4_		
	LTD_4_		
	LTE_4_		

Anti-inflammatory effects of the n-3 fatty-acid-derived eicosanoids			

	EPA and DHA (n-3) derived eicosanoids	Physiological effects	Organs or cells

Prostaglandins	PGD_3_		
	PGE_3_	Antiarrhythmic	Vessels
	PGF_3_		
	PGI_3_	Antiarrhythmic	Vessels

Thromboxanes	TXA_3_	Antiaggregation	Platelets
	TXB_3_	Antiaggregation	Platelets

Leukotrienes	LTA_5_		
	LTB_5_	Anti-inflammatory	Leukocytes
	LTC_5_		
	LTD_5_		
	LTE_5_		

Resolvins	RVE_1_	Antiaggregation Anti-inflammatory	Platelets Dendritic cells
	RVD	Anti-inflammatory	

Neuroprotectin	NPD_1_	Anti-inflammatory Antiapoptotic Decreases oxidative stress	Retina (photoreceptor cells) and brain

Data elaborated from [21, 37–39].

## References

[1] Calder PC (2008). Polyunsaturated fatty acids, inflammatory processes and inflammatory bowel diseases. *Molecular Nutrition and Food Research*.

[2] Wall R, Ross RP, Fitzgerald GF, Stanton C (2010). Fatty acids from fish: the anti-inflammatory potential of long-chain omega-3 fatty acids. *Nutrition Reviews*.

[3] Calder PC (2009). Polyunsaturated fatty acids and inflammatory processes: new twists in an old tale. *Biochimie*.

[4] Olivier MC, Vanessa L, Isabelle A (2011). Why and how meet n-3 PUFA dietary recommendations?. *Gastroenterology Research and Practice*.

[5] Simopoulos AP (2011). Evolutionary aspects of Diet: the omega-6/omega-3 ratio and the brain. *Molecular Neurobiology*.

[6] Linseisen J, Welch AA, Ocké M (2009). Dietary fat intake in the European Prospective Investigation into Cancer and Nutrition: results from the 24-h dietary recalls. *European Journal of Clinical Nutrition*.

[7] Eaton SB, Konner MJ, Cordain L (2010). Diet-dependent acid load, Paleolithic nutrition, and evolutionary health promotion. *The American Journal of Clinical Nutrition*.

[8] Simopoulos AP (2001). N-3 fatty acids and human health: defining strategies for public policy. *Lipids*.

[9] Anderson BM, Ma D (2009). Are all n-3 polyunsaturated fatty acids created equal?. *Lipids in Health and Disease*.

[10] Corsinovi L, Biasi F, Poli G, Leonarduzzi G, Isaia G (2011). Dietary lipids and their oxidized products in Alzheimer's disease. *Molecular Nutrition and Food Research*.

[11] Hassan STBS, Hanachi P (2009). Dietary patterns and the metabolic syndrome in middle aged women, Babol, Iran. *Asia Pacific Journal of Clinical Nutrition*.

[12] Ruidavets JB, Bongard V, Dallongeville J (2007). High consumptions of grain, fish, dairy products and combinations of these are associated with a low prevalence of metabolic syndrome. *Journal of Epidemiology and Community Health*.

[13] Das UN (2006). Essential fatty acids: biochemistry, physiology and pathology. *Biotechnology Journal*.

[14] Hughes CL, Dhiman TR (2002). Dietary compounds in relation to dietary diversity and human health. *Journal of Medicinal Food*.

[15] Uauy R, Dangour AD (2006). Nutrition in brain development and aging: role of essential fatty acids. *Nutrition Reviews*.

[16] Lauretani F, Semba RD, Bandinelli S (2008). Plasma polyunsaturated fatty acids and the decline of renal function. *Clinical Chemistry*.

[17] El-Badry AM, Graf R, Clavien PA (2007). Omega 3—omega 6: what is right for the liver?. *Journal of Hepatology*.

[18] Benatti P, Peluso G, Nicolai R, Calvani M (2004). Polyunsaturated fatty acids: biochemical, nutritional and epigenetic properties. *Journal of the American College of Nutrition*.

[19] Stoffel W, Holz B, Jenke B (2008). Δ6-Desaturase (FADS2) deficiency unveils the role of *ω*3- and *ω*6-polyunsaturated fatty acids. *The EMBO Journal*.

[20] Moon YA, Hammer RE, Horton JD (2009). Deletion of ELOVL5 leads to fatty liver through activation of SREBP-1c in mice. *Journal of Lipid Research*.

[21] Schmitz G, Ecker J (2008). The opposing effects of n-3 and n-6 fatty acids. *Progress in Lipid Research*.

[22] Stroud CK, Nara TY, Roqueta-Rivera M (2009). Disruption of FADS2 gene in mice impairs male reproduction and causes dermal and intestinal ulceration. *Journal of Lipid Research*.

[23] Araya J, Rodrigo R, Pettinelli P, Araya AV, Poniachik J, Videla LA (2010). Decreased liver fatty Acid delta-6 and delta-5 desaturase activity in obese patients. *Obesity*.

[24] Brenner RR (2003). Hormonal modulation of delta6 and delta5 desaturases: case of diabetes. *Prostaglandins, Leukotrienes and Essential Fatty Acids*.

[25] Brenner RR (1981). Nutritional and hormonal factors influencing desaturation of essential fatty acids. *Progress in Lipid Research*.

[26] Das UN (2006). Biological significance of essential fatty acids. *Journal of Association of Physicians of India*.

[27] Simopoulos AP (2002). Omega-3 fatty acids in inflammation and autoimmune diseases. *Journal of the American College of Nutrition*.

[28] Zadravec D, Tvrdik P, Guillou H (2011). ELOVL2 controls the level of n-6 28:5 and 30:5 fatty acids in testis, a prerequisite for male fertility and sperm maturation in mice. *Journal of Lipid Research*.

[29] Russo GL (2009). Dietary n-6 and n-3 polyunsaturated fatty acids: from biochemistry to clinical implications in cardiovascular prevention. *Biochemical Pharmacology*.

[30] Wang D, Dubois RN (2010). Eicosanoids and cancer. *Nature Reviews Cancer*.

[31] Ricciotti E, Fitzgerald GA (2011). Prostaglandins and inflammation. *Arteriosclerosis, Thrombosis, and Vascular Biology*.

[32] Tai CC, Ding ST (2010). N-3 polyunsaturated fatty acids regulate lipid metabolism through several inflammation mediators: mechanisms and implications for obesity prevention. *Journal of Nutritional Biochemistry*.

[33] de Batlle J Association between [Omega] 3 and [Omega] 6 fatty acid intakes and serum inflammatory markers in COPD.

[34] Poudel-Tandukar K, Nanri A, Matsushita Y (2009). Dietary intakes of *α*-linolenic and linoleic acids are inversely associated with serum C-reactive protein levels among Japanese men. *Nutrition Research*.

[35] Yoneyama S, Miura K, Sasaki S (2007). Dietary intake of fatty acids and serum C-reactive protein in Japanese. *Journal of Epidemiology*.

[36] Fritsche KL (2008). Too much linoleic acid promotes inflammation—doesn't it?. *Prostaglandins, Leukotrienes and Essential Fatty Acids*.

[37] Harizi H, Corcuff JB, Gualde N (2008). Arachidonic-acid-derived eicosanoids: roles in biology and immunopathology. *Trends in Molecular Medicine*.

[38] Bazan NG (2009). Cellular and molecular events mediated by docosahexaenoic acid-derived neuroprotectin D1 signaling in photoreceptor cell survival and brain protection. *Prostaglandins, Leukotrienes and Essential Fatty Acids*.

[39] Fredman G, Serhan CN (2011). Specialized proresolving mediator targets for RvE1 and RvD1 in peripheral blood and mechanisms of resolution. *The Biochemical Journal*.

[40] Levin G, Duffin KL, Obukowicz MG (2002). Differential metabolism of dihomo-*γ*-linolenic acid and arachidonic acid by cyclo-oxygenase-1 and cyclo-oxygenase-2: implications for cellular synthesis of prostaglandin E-1 and prostaglandin E-2. *Biochemical Journal*.

[41] Bagga D, Wang L, Farias-Eisner R, Glaspy JA, Reddy ST (2003). Differential effects of prostaglandin derived from *ω*-6 and *ω*-3 polyunsaturated fatty acids on COX-2 expression and IL-6 secretion. *Proceedings of the National Academy of Sciences of the United States of America*.

[42] Pulichino AM, Rowland S, Wu T (2006). Prostacyclin antagonism reduces pain and inflammation in rodent models of hyperalgesia and chronic arthritis. *Journal of Pharmacology and Experimental Therapeutics*.

[43] Kojima F, Kato S, Kawai S (2005). Prostaglandin E synthase in the pathophysiology of arthritis. *Fundamental and Clinical Pharmacology*.

[44] Tilley SL, Coffman TM, Koller BH (2001). Mixed messages: modulation of inflammation and immune responses by prostaglandins and thromboxanes. *Journal of Clinical Investigation*.

[45] Peters-Golden M, Henderson WR (2007). Leukotrienes. *New England Journal of Medicine*.

[46] Robinson JG, Stone NJ (2006). Antiatherosclerotic and antithrombotic effects of omega-3 fatty acids. *American Journal of Cardiology*.

[47] Hata AN, Breyer RM (2004). Pharmacology and signaling of prostaglandin receptors: multiple roles in inflammation and immune modulation. *Pharmacology and Therapeutics*.

[48] Narumiya S, FitzGerald GA (2001). Genetic and pharmacological analysis of prostanoid receptor function. *Journal of Clinical Investigation*.

[49] Ide T, Egan K, Bell-Parikh LC, FitzGerald GA (2003). Activation of nuclear receptors by prostaglandins. *Thrombosis Research*.

[50] Arima M, Fukuda T (2011). Prostaglandin D_2_ and T_H_2 inflammation in the pathogenesis of bronchial asthma. *The Korean Journal of Internal Medicine*.

[51] Pettipher R, Hansel TT, Armer R (2007). Antagonism of the prostaglandin D2 receptors DP1 and CRTH2 as an approach to treat allergic diseases. *Nature Reviews Drug Discovery*.

[52] Lundeen KA, Sun B, Karlsson L, Fourie AM (2006). Leukotriene B4 receptors BLT1 and BLT2: expression and function in human and murine mast cells. *Journal of Immunology*.

[53] Jupp J, Hillier K, Elliott DH (2007). Colonic expression of leukotriene-pathway enzymes in inflammatory bowel diseases. *Inflammatory Bowel Diseases*.

[54] Funk CD (2001). Prostaglandins and leukotrienes: advances in eicosanoid biology. *Science*.

[55] Serhan CN, Savill J (2005). Resolution of inflammation: the beginning programs the end. *Nature Immunology*.

[56] Chawla A, Repa JJ, Evans RM, Mangelsdorf DJ (2001). Nuclear receptors and lipid physiology: opening the X-files. *Science*.

[57] Bensinger SJ, Tontonoz P (2008). Integration of metabolism and inflammation by lipid-activated nuclear receptors. *Nature*.

[58] Issemann I, Green S (1990). Activation of a member of the steroid hormone receptor superfamily by peroxisome proliferators. *Nature*.

[59] Couet C, Delarue J, Ritz P, Antoine JM, Lamisse F (1997). Effect of dietary fish oil on body fat mass and basal fat oxidation in healthy adults. *International Journal of Obesity*.

[60] Power GW, Newsholme EA (1997). Dietary fatty acids influence the activity and metabolic control of mitochondrial carnitine palmitoyltransferase I in rat heart and skeletal muscle. *Journal of Nutrition*.

[61] Hu FB, Stampfer MJ, Manson JE (1999). Dietary intake of *α*-linolenic acid and risk of fatal ischemic heart disease among women. *American Journal of Clinical Nutrition*.

[62] Mori TA, Bao DQ, Burke V, Puddey IB, Watts GF, Beilin LJ (1999). Dietary fish as a major component of a weight-loss diet: effect on serum lipids, glucose, and insulin metabolism in overweight hypertensive subjects. *American Journal of Clinical Nutrition*.

[63] Chakravarthy MV, Lodhi IJ, Yin L (2009). Identification of a physiologically relevant endogenous ligand for PPAR*α* in liver. *Cell*.

[64] Yoon MJ, Gha YL, Chung JJ, Young HA, Seung HH, Jae BK (2006). Adiponectin increases fatty acid oxidation in skeletal muscle cells by sequential activation of AMP-activated protein kinase, p38 mitogen-activated protein kinase, and peroxisome proliferator—activated receptor*α*. *Diabetes*.

[65] Fujita K, Maeda N, Sonoda M (2008). Adiponectin protects against angiotensin II-induced cardiac fibrosis through activation of PPAR-*α*. *Arteriosclerosis, Thrombosis, and Vascular Biology*.

[66] Perkins ND (2007). Integrating cell-signalling pathways with NF-*κ*B and IKK function. *Nature Reviews Molecular Cell Biology*.

[67] Zhao Y, Joshi-Barve S, Barve S, Chen LH (2004). Eicosapentaenoic acid prevents LPS-induced TNF-*α* expression by preventing NF-*κ*B activation. *Journal of the American College of Nutrition*.

[68] Weldon SM, Mullen AC, Loscher CE, Hurley LA, Roche HM (2007). Docosahexaenoic acid induces an anti-inflammatory profile in lipopolysaccharide-stimulated human THP-1 macrophages more effectively than eicosapentaenoic acid. *Journal of Nutritional Biochemistry*.

[69] de Caterina R, Spiecker M, Solaini G (1999). The inhibition of endothelial activation by unsaturated fatty acids. *Lipids*.

[70] Lepley RA, Fitzpatrick FA (1998). 5-lipoxygenase compartmentalization in granulocytic cells is modulated by an internal bipartite nuclear localizing sequence and nuclear factor *κ*B complex formation. *Archives of Biochemistry and Biophysics*.

[71] Soberman RJ, Christmas P (2003). The organization and consequences of eicosanoid signaling. *Journal of Clinical Investigation*.

[72] Ecker J, Langmann T, Moehle C, Schmitz G (2007). Isomer specific effects of Conjugated Linoleic Acid on macrophage ABCG1 transcription by a SREBP-1c dependent mechanism. *Chemistry and Physics of Lipids*.

[73] Xu J, Cho H, O'Malley S, Park JHY, Clarke SD (2002). Dietary polyunsaturated fats regulate rat liver sterol regulatory element binding proteins-1 and -2 in three distinct stages and by different mechanisms. *Journal of Nutrition*.

[74] Teran-Garcia M, Adamson AW, Yu G (2007). Polyunsaturated fatty acid suppression of fatty acid synthase (FASN): evidence for dietary modulation of NF-Y binding to the Fasn promoter by SREBP-1c. *Biochemical Journal*.

[75] Xu J, Nakamura MT, Cho HP, Clarke SD (1999). Sterol regulatory element binding protein-1 expression is suppressed by dietary polyunsaturated fatty acids. A mechanism for the coordinate suppression of lipogenic genes by polyunsaturated fats. *Journal of Biological Chemistry*.

[76] Xu J, Teran-Garcia M, Park JHY, Nakamura MT, Clarke SD (2001). Polyunsaturated fatty acids suppress hepatic sterol regulatory element-binding protein-1 expression by accelerating transcript decay. *Journal of Biological Chemistry*.

[77] Howell G, Deng X, Yellaturu C (2009). N-3 polyunsaturated fatty acids suppress insulin-induced SREBP-1c transcription via reduced trans-activating capacity of LXR*α*. *Biochimica et Biophysica Acta: Molecular and Cell Biology of Lipids*.

[78] Pachikian BD, Essaghir A, Demoulin J-B (2011). Hepatic n-3 polyunsaturated fatty acid depletion promotes steatosis and insulin resistance in mice: genomic analysis of cellular targets. *PLoS ONE*.

[79] Pawar A, Botolin D, Mangelsdorf DJ, Jump DB (2003). The role of liver X receptor-*α* in the fatty acid regulation of hepatic gene expression. *Journal of Biological Chemistry*.

[80] Yoshikawa T, Ide T, Shimano H (2003). Cross-talk between peroxisome proliferator-activated receptor (PPAR) *α* and liver X receptor (LXR) in nutritional regulation of fatty acid metabolism. I. PPARS suppress sterol regulatory element binding protein-1c promoter through inhibition of LXR signaling. *Molecular Endocrinology*.

[81] Byrne C, Olufad R, Bruce KD, Cagampang FR, Ahmed MH (2009). Metabolic disturbances in non-alcoholic fatty liver disease. *Clinical Science*.

[82] Byrne CD (2010). Fatty liver: role of inflammation and fatty acid nutrition. *Prostaglandins, Leukotrienes and Essential Fatty Acids*.

[83] Erickson SK (2009). Nonalcoholic fatty liver disease. *Journal of Lipid Research*.

[84] Videla LA, Rodrigo R, Araya J, Poniachik J (2006). Insulin resistance and oxidative stress interdependency in non-alcoholic fatty liver disease. *Trends in Molecular Medicine*.

[85] Larter CZ, Yeh MM, Cheng J (2008). Activation of peroxisome proliferator-activated receptor *α* by dietary fish oil attenuates steatosis, but does not prevent experimental steatohepatitis because of hepatic lipoperoxide accumulation. *Journal of Gastroenterology and Hepatology*.

[86] Araya J, Rodrigo R, Videla LA (2004). Increase in long-chain polyunsaturated fatty acid n-6/n-3 ratio in relation to hepatic steatosis in patients with non-alcoholic fatty liver disease. *Clinical Science*.

[87] Spadaro L, Magliocco O, Spampinato D (2008). Effects of n-3 polyunsaturated fatty acids in subjects with nonalcoholic fatty liver disease. *Digestive and Liver Disease*.

[88] Zivkovic AM, German JB, Sanyal AJ (2007). Comparative review of diets for the metabolic syndrome: implications for nonalcoholic fatty liver disease. *American Journal of Clinical Nutrition*.

[89] Jump DB (2008). N-3 polyunsaturated fatty acid regulation of hepatic gene transcription. *Current Opinion in Lipidology*.

[90] Ip E, Farrell G, Hall P, Robertson G, Leclercq I (2004). Administration of the potent PPAR*α* agonist, Wy-14,643, reverses nutritional fibrosis and steatohepatitis in mice. *Hepatology*.

[91] Ip E, Farrell GC, Robertson G, Hall P, Kirsch R, Leclercq I (2003). Central role of PPAR*α*-dependent hepatic lipid turnover in dietary steatohepatitis in mice. *Hepatology*.

[92] Lindén D, Lindberg K, Oscarsson J (2002). Influence of peroxisome proliferator-activated receptor *α* agonists on the intracellular turnover and secretion of apolipoprotein (Apo) B-100 and ApoB-48. *Journal of Biological Chemistry*.

[93] Carlsson L, Lindén D, Jalouli M, Oscarsson J (2001). Effects of fatty acids and growth hormone on liver fatty acid binding protein and PPAR*α* in rat liver. *American Journal of Physiology: Endocrinology and Metabolism*.

[94] Savage DB, Semple RK (2010). Recent insights into fatty liver, metabolic dyslipidaemia and their links to insulin resistance. *Current Opinion in Lipidology*.

[95] Kersten S, Seydoux J, Peters JM, Gonzalez FJ, Desvergne B, Wahli W (1999). Peroxisome proliferator-activated receptor *α* mediates the adaptive response to fasting. *Journal of Clinical Investigation*.

[96] Masterton G, Plevris J, Hayes P (2010). Review article: omega-3 fatty acids—a promising novel therapy for non-alcoholic fatty liver disease. *Alimentary Pharmacology and Therapeutics*.

[97] Yahagi N, Shimano H, Hasty AH (1999). A crucial role of sterol regulatory element-binding protein-1 in the regulation of lipogenic gene expression by polyunsaturated fatty acids. *Journal of Biological Chemistry*.

[98] Malaisse WJ, Bulur N, Zhang Y (2009). The metabolic syndrome of *ω*3-depleted rats. I. Liver data. *International Journal of Molecular Medicine*.

[99] Videla LA, Rodrigo R, Araya J, Poniachik J (2004). Oxidative stress and depletion of hepatic long-chain polyunsaturated fatty acids may contribute to nonalcoholic fatty liver disease. *Free Radical Biology and Medicine*.

[100] Narasimhan S, Gokulakrishnan K, Sampathkumar R (2010). Oxidative stress is independently associated with non-alcoholic fatty liver disease (NAFLD) in subjects with and without type 2 diabetes. *Clinical Biochemistry*.

[101] Chalasani N, Deeg MA, Crabb DW (2004). Systemic levels of lipid peroxidation and its metabolic and dietary correlates in patients with nonalcoholic steatohepatitis. *American Journal of Gastroenterology*.

[102] Yesilova Z, Yaman H, Oktenli C (2005). Systemic markers of lipid peroxidation and antioxidants in patients with nonalcoholic fatty liver disease. *American Journal of Gastroenterology*.

[103] Seki S, Kitada T, Yamada T, Sakaguchi H, Nakatani K, Wakasa K (2002). In situ detection of lipid peroxidation and oxidative DNA damage in non-alcoholic fatty liver diseases. *Journal of Hepatology*.

[104] Feldstein AE, Werneburg NW, Canbay A (2004). Free fatty acids promote hepatic lipotoxicity by stimulating TNF-*α* expression via a lysosomal pathway. *Hepatology*.

[105] Allard JP, Aghdassi E, Mohammed S (2008). Nutritional assessment and hepatic fatty acid composition in non-alcoholic fatty liver disease (NAFLD): a cross-sectional study. *Journal of Hepatology*.

[106] Sevanian A, Hochstein P (1985). Mechanisms and consequences of lipid peroxidation in biological systems. *Annual Review of Nutrition*.

[107] Lombardo YB, Chicco AG (2006). Effects of dietary polyunsaturated n-3 fatty acids on dyslipidemia and insulin resistance in rodents and humans. A review. *Journal of Nutritional Biochemistry*.

[108] Vuppalanchi R, Cummings OW, Saxena R (2007). Relationship among histologic, radiologic, and biochemical assessments of hepatic steatosis: a study of human liver samples. *Journal of Clinical Gastroenterology*.

[109] Montecucco F, Mach F (2009). Atherosclerosis is an Inflammatory Disease. *Seminars in Immunopathology*.

[110] Das UN (2007). A defect in the activity of *δ*6 and *δ*5 desaturases may be a factor in the initiation and progression of atherosclerosis. *Prostaglandins, Leukotrienes and Essential Fatty Acids*.

[111] Bang HO, Dyerberg J (1976). Lipid metabolism and ischemic heart disease in Greenland Eskimos. *Acta Medica Scandinavica*.

[112] Menotti A, Kromhout D, Blackburn H, Fidanza F, Buzina R, Nissinen A (1999). Food intake patterns and 25-year mortality from coronary heart disease: cross-cultural correlations in the Seven Countries Study. *European Journal of Epidemiology*.

[113] Holub BJ (2002). Clinical nutrition: 4. Omega-3 fatty acids in cardiovascular care. *Canadian Medical Association Journal*.

[114] Lemaitre RN, King IB, Mozaffarian D, Kuller LH, Tracy RP, Siscovick DS (2003). n-3 polyunsaturated fatty acids, fatal ischemic heart disease, and nonfatal myocardial infarction in older adults: the Cardiovascular Health Study. *American Journal of Clinical Nutrition*.

[115] He K, Song Y, Daviglus ML (2004). Accumulated evidence on fish consumption and coronary heart disease mortality—a meta-analysis of cohort studies. *Circulation*.

[116] Mozaffarian D, Geelen A, Brouwer IA, Geleijnse JM, Zock PL, Katan MB (2005). Effect of fish oil on heart rate in humans—a meta-analysis of randomized controlled trials. *Circulation*.

[117] Sellers MM, Stallone JN (2008). Sympathy for the devil: the role of thromboxane in the regulation of vascular tone and blood pressure. *American Journal of Physiology: Heart and Circulatory Physiology*.

[118] Kobayashi T, Tahara Y, Matsumoto M (2004). Roles of thromboxane *A* ~ 2 and prostacyclin in the development of atherosclerosis in apoE-deficient mice. *Journal of Clinical Investigation*.

[119] Harris WS, Mozaffarian D, Rimm E (2009). Omega-6 fatty acids and risk for cardiovascular disease: a science advisory from the American Heart Association nutrition subcommittee of the council on nutrition, physical activity, and metabolism; council on cardiovascular nursing; and council on epidemiology and prevention. *Circulation*.

[120] Maingrette F, Renier G (2005). Linoleic acid increases lectin-like oxidized LDL receptor-1 (LOX-1) expression in human aortic endothelial cells. *Diabetes*.

[121] Simopoulos AP (2008). The importance of the omega-6/omega-3 fatty acid ratio in cardiovascular disease and other chronic diseases. *Experimental Biology and Medicine*.

[122] Prescott J, Owens D, Collins P, Johnson A, Tomkin GH (1999). The fatty acid distribution in low density lipoprotein in diabetes. *Biochimica et Biophysica Acta: Molecular and Cell Biology of Lipids*.

[123] Regnstrom J, Nilsson J, Tornvall P, Landou C, Hamsten A (1992). Susceptibility to low-density lipoprotein oxidation and coronary atherosclerosis in man. *Lancet*.

[124] Liou YA, King DJ, Zibrik D, Innis SM (2007). Decreasing linoleic acid with constant *α*-linolenic acid in dietary fats increases (n-3) eicosapentaenoic acid in plasma phospholipids in healthy men. *Journal of Nutrition*.

[125] Lucendo AJ, de Rezende LC (2009). Importance of nutrition in inflammatory bowel disease. *World Journal of Gastroenterology*.

[126] Cashman KD, Shanahan F (2003). Is nutrition an aetiological factor for inflammatory bowel disease?. *European Journal of Gastroenterology and Hepatology*.

[127] Kromann N, Green A (1980). Epidemiological studies in the Upernavik district, Greenland. *Acta Medica Scandinavica*.

[128] Bang H, Dyerberg J, Sinclair HM (1980). The composition of the Eskimo food in north Western Greenland. *American Journal of Clinical Nutrition*.

[129] Shimizu T, Fujii T, Suzuki R (2003). Effects of highly purified eicosapentaenoic acid on erythrocyte fatty acid composition and leukocyte and colonic mucosa leukotriene B4 production in children with ulcerative colitis. *Journal of Pediatric Gastroenterology and Nutrition*.

[130] Hawthorne AB, Daneshmend TK, Hawkey a CJ (1992). Treatment of ulcerative colitis with fish oil supplementation—a prospective 12 month randomised controlled trial. *Gut*.

[131] Chapkin RS, Davidson LA, Ly L, Weeks BR, Lupton JR, McMurray DN (2007). Immunomodulatory effects of (n-3) fatty acids: putative link to inflammation and colon cancer. *Journal of Nutrition*.

[132] Sakamoto N, Kono S, Wakai K (2005). Dietary risk factors for inflammatory bowel disease: a multicenter case-control study in Japan. *Inflammatory Bowel Diseases*.

[133] Shoda R, Matsueda K, Yamato S, Umeda N (1996). Epidemiologic analysis of Crohn disease in Japan: increased dietary intake of n-6 polyunsaturated fatty acids and animal protein relates to the increased incidence of Crohn disease in Japan. *American Journal of Clinical Nutrition*.

[134] Sharon P, Stenson WF (1984). enhanced synthesis of leukotriene-B4 by colonic mucosa in inflammatory bowel-disease. *Gastroenterology*.

[135] Belluzzi A, Brignola C, Campieri M, Pera A, Boschi S, Miglioli M (1996). Effect of an enteric-coated fish-oil preparation on relapses in Crohn's disease. *New England Journal of Medicine*.

[136] Ferrucci L, Cherubini A, Bandinelli S (2006). Relationship of plasma polyunsaturated fatty acids to circulating inflammatory markers. *Journal of Clinical Endocrinology and Metabolism*.

[137] Sijben JWC, Calder PC (2007). Differential immunomodulation with long-chain n-3 PUFA in health and chronic disease. *Proceedings of the Nutrition Society*.

[138] Calder PC (2008). PUFA, inflammatory processes and rheumatoid arthritis. *Proceedings of the Nutrition Society*.

[139] Honda T, Segi-Nishida E, Miyachi Y, Narumiya S (2006). Prostacyclin-IP signaling and prostaglandin E2-EP2/EP4 signaling both mediate joint inflammation in mouse collagen-induced arthritis. *Journal of Experimental Medicine*.

[140] Curtis CL, Hughes CE, Flannery CR, Little CB, Harwood JL, Caterson B (2000). n-3 Fatty acids specifically modulate catabolic factors involved in articular cartilage degradation. *Journal of Biological Chemistry*.

[141] Adam O, Beringer C, Kless T (2003). Anti-inflammatory effects of a low arachidonic acid diet and fish oil in patients with rheumatoid arthritis. *Rheumatology International*.

[142] Björkhem I, Cedazo-Minguez A, Leoni V, Meaney S (2009). Oxysterols and neurodegenerative diseases. *Molecular Aspects of Medicine*.

[143] Whelan J (2008). (n-6) and (n-3) polyunsaturated fatty acids and the aging brain: food for thought. *Journal of Nutrition*.

[144] Rothman SM, Mattson MP (2010). Adverse stress, hippocampal networks, and Alzheimer's disease. *NeuroMolecular Medicine*.

[145] Lee HP, Zhu X, Casadesus G (2010). Antioxidant approaches for the treatment of Alzheimers disease. *Expert Review of Neurotherapeutics*.

[146] Farooqui AA, Horrocks LA, Farooqui T (2007). Modulation of inflammation in brain: a matter of fat. *Journal of Neurochemistry*.

[147] Tassoni D, Kaur G, Weisinger RS, Sinclair AJ (2008). The role of eicosanoids in the brain. *Asia Pacific Journal of Clinical Nutrition*.

[148] Calon F, Lim GP, Morihara T (2005). Dietary n-3 polyunsaturated fatty acid depletion activates caspases and decreases NMDA receptors in the brain of a transgenic mouse model of Alzheimer's disease. *European Journal of Neuroscience*.

[149] Schaefer EJ, Bongard V, Beiser AS (2006). Plasma phosphatidylcholine docosahexaenoic acid content and risk of dementia and Alzheimer disease: the Framingham Heart Study. *Archives of Neurology*.

[150] Hashimoto M, Hossain S, Shimada T (2002). Docosahexaenoic acid provides protection from impairment of learning ability in Alzheimer's disease model rats. *Journal of Neurochemistry*.

[151] Lim GP, Calon F, Morihara T (2005). A diet enriched with the omega-3 fatty acid docosahexaenoic acid reduces amyloid burden in an aged Alzheimer mouse model. *Journal of Neuroscience*.

[152] Dangour A, Allen E, Elbourne D, Fletcher A, Richards M, Uauy R (2009). Fish consumption and cognitive function among older people in the UK: baseline data from the OPAL study. *Journal of Nutrition, Health and Aging*.

[153] Freeman MP, Hibbeln JR, Wisner KL (2006). Omega-3 fatty acids: evidence basis for treatment and future research in psychiatry. *Journal of Clinical Psychiatry*.

[154] Freemantle E, Vandal M, Tremblay-Mercier J (2006). Omega-3 fatty acids, energy substrates, and brain function during aging. *Prostaglandins, Leukotrienes and Essential Fatty Acids*.

[155] Samieri C, Féart C, Letenneur L (2008). Low plasma eicosapentaenoic acid and depressive symptomatology are independent predictors of dementia risk. *American Journal of Clinical Nutrition*.

[156] Dinan T, Siggins L, Scully P, O'Brien S, Ross P, Stanton C (2009). Investigating the inflammatory phenotype of major depression: focus on cytokines and polyunsaturated fatty acids. *Journal of Psychiatric Research*.

